# Comparison of three point-of-care testing devices to detect hemostatic changes in adult elective cardiac surgery: a prospective observational study

**DOI:** 10.1186/1471-2253-14-80

**Published:** 2014-09-22

**Authors:** Aurora Espinosa, Roar Stenseth, Vibeke Videm, Hilde Pleym

**Affiliations:** 1 Department of Immunology and Transfusion Medicine, St. Olav University Hospital, Trondheim, Norway; 2 Department of Cardiothoracic Anesthesia and Intensive Care Medicine, St. Olav University Hospital, Trondheim, Norway; 3 Department of Circulation and Medical Imaging, Norwegian University of Science and Technology, Trondheim, Norway; 4 Department of Laboratory Medicine, Children’s and Women’s Health, Norwegian University of Science and Technology, Trondheim, Norway; 5 Clinic of Anaesthesia and Intensive Care, St. Olav University Hospital, Trondheim, Norway

**Keywords:** Blood, Cardiac surgery, Cardiopulmonary bypass (CPB), Coagulation, Thromboelastography, Thromboelastometry

## Abstract

**Background:**

Bleeding complications in cardiac surgery may lead to increased morbidity and mortality. Traditional blood coagulation tests are not always suitable to detect rapid changes in the patient's coagulation status. Point-of-care instruments such as the TEG (thromboelastograph) and RoTEM (thromboelastometer) have been shown to be useful as a guide for the clinician in the choice of blood products and they may lead to a reduction in the need for blood transfusion, contributing to better patient blood management.

**Methods:**

The purpose of this study was to evaluate the ability of the TEG, RoTEM and Sonoclot instruments to detect changes in hemostasis in elective cardiac surgery with cardiopulmonary bypass and to investigate possible correlations between variables from these three instruments and routine hematological coagulation tests. Blood samples from thirty-five adult patients were drawn before and after surgery and analyzed in TEG, RoTEM, Sonoclot and routine coagulation tests. Data were compared using repeated measures analysis of variance and Pearson's test for linear correlation.

**Results:**

We found significant changes for all TEG variables after surgery, for three of the RoTEM variables, and for one variable from the Sonoclot. There were significant correlations postoperatively between plasma fibrinogen levels and variables from the three instruments.

**Conclusions:**

TEG and RoTEM may be used to detect changes in hemostasis following cardiac surgery with CPB. Sonoclot seems to be less suitable to detect such changes. Variables from the three instruments correlated with plasma fibrinogen and could be used to monitor treatment with fibrinogen concentrate.

## Background

Excessive bleeding in cardiac surgery occurs in as much as 20% of patients. It is associated with increased morbidity and mortality, mainly caused by need for reexploration and transfusion of blood products [1-6]. In 15-50% of the patients undergoing reexploration, a surgical cause of bleeding cannot be found [7,8] and it is assumed to be of microvascular origin. The pathogenesis of microvascular bleeding in cardiac surgery is multifactorial and may be related to changes in the hemostatic system associated with increased age, preoperative medication with platelet and/or coagulation inhibitors, and transient platelet dysfunction associated with cardiopulmonary bypass (CPB) [6-12]. The use of CPB induces a systemic inflammatory response with activation of both the coagulation and fibrinolytic systems, followed by coagulopathy caused by factor consumption and transiently reduced platelet count and function [13,14].

To date, there is no “gold standard” test for the assessment of microvascular bleeding in cardiac surgery. Perioperative monitoring of the hemostatic process by a combination of conventional coagulation tests may be helpful in diagnosing possible causes of bleeding [15,16]. However, the value of these tests has been questioned, mainly because they are time-consuming, performed in plasma and do not add information about platelet function or the contribution of the cellular components [17,18].

In the last decade, viscoelastic point-of-care (POC) coagulation instruments such as the thromboelastograph (TEG), the thromboelastometer (RoTEM) and the Sonoclot have been increasingly used in clinical practice for perioperative monitoring of excessive bleeding in cardiac surgery [18-20]. These POC instruments provide information about the quality and dynamics of the clot, are relatively simple to operate and can be used close to the patient. The results are usually ready for interpretation within 15–30 minutes. Guidelines for interpretation and use of POC instruments in the management of perioperative coagulopathy and transfusion algorithms have been developed [21]. These instruments may be helpful as a guide for transfusion therapy and to decide whether to reexplore a patient or not [17-19,22].

Although with some differences, these instruments are based on similar principles and provide output in the form of a similar graphical tracing. However, it is still not clear whether TEG, RoTEM and Sonoclot provide identical information. The performance of different POC instruments in cardiac surgery has been evaluated in previous studies [22-24] but, to our knowledge, TEG, RoTEM and Sonoclot instruments have not previously been directly compared in the same group of cardiac surgery patients.

The primary objective of this study was to evaluate the comparability of the results obtained from the TEG, RoTEM and Sonoclot instruments by assessing their ability to detect changes in hemostasis caused by CPB. In addition, we also investigated whether variables from the TEG, RoTEM and Sonoclot analyses were correlated to results from a selection of conventional coagulation tests.

## Methods

### Study design and patients

This study was a prospective observational study. Thirty-five patients (22 men and 13 women) scheduled for elective cardiac surgery with the use of CPB were included after giving written informed consent. All patients were treated with aspirin until the day before surgery. Patients who had been treated with clopidogrel within five days prior to surgery were not considered eligible to the study.

### Group assignment and procedures

Patients with unstable angina received low molecular weight heparin preoperatively. Tranexamic acid 30 mg/kg was given to all patients prior to CPB according to departmental routines. Before CPB, the patients received heparin 300 U/kg (Leo, Copenhagen, Denmark) through a central venous line to achieve a kaolin activated clotting time (ACT) (Medtronic Blood Management, Parker, CO, USA) of > 480 seconds. Additional heparin was given when needed to keep the ACT above the target. During CPB, the ACT was monitored every 20 minutes. The perfusion circuit was primed with 1800 mL of Ringer’s acetate solution with 7500 U of heparin. A membrane oxygenator without heparin coating was used.

Cold antegrade and/or retrograde blood or crystalloid cardioplegia and moderate hypothermia to 34°C were used during CPB. Cardiotomy suction was used while the patients were fully anticoagulated, and the blood was returned to the patients without centrifugation. The patients were warmed to a rectal temperature of at least 36°C before termination of CPB. After CPB, protamine sulphate was given to achieve an ACT within 10% of the baseline value. Blood remaining in the CPB circuit was collected and transfused to the patient. Postoperatively, transfusions of packed red cells were given when the hemoglobin concentration was < 8.5 – 9.0 g/dL. Persistent postoperative bleeding of more than 200 mL/h was treated with infusions of desmopressin, fresh frozen plasma and/or platelet concentrates, or reexploration at the discretion of the attending clinician.

To identify possible hemostatic changes caused by CPB in the TEG, RoTEM, Sonoclot and conventional coagulation tests, blood samples were collected at three different time points: preoperatively (immediately before anesthesia induction), and at 1 and 24 hours postoperatively. Blood samples for TEG and RoTEM analyses were drawn into a tube containing citrate and analyzed within 1 hour, after recalcification with 20 μL CaCl_2_. Blood samples for Sonoclot analysis were drawn into syringes without additives and transferred to the Sonoclot cuvette for immediate analysis. All the tests for the TEG, RoTEM and Sonoclot were performed by the same investigator (AE), and all Sonoclot tracings were interpreted by a single investigator (HP). The operating principles of the TEG (Haemoscope Corp., Niles, IL, USA), RoTEM (Pentapharm GmbH, Munich, Germany) and the Sonoclot analyzer (Sienco Inc., Arvada, CO, USA) are described in detail by Ganter and Hofer [17]. Nomenclature and explanation for the different TEG, RoTEM and Sonoclot variables, as well as the reference values are given in Table 1.

**Table 1:** Nomenclature, variable definitions and reference ranges of the TEG, RoTEM and Sonoclot instruments

	**TEG**	**RoTEM**	**Sonoclot**
Clot time	R (Reaction time)	CT (Clotting time)	-
Time to 2 mm amplitude	(4–8 min.)	-EXTEM CT (42–74 sec)*	
		-INTEM CT (137–246 sec)	
Clot kinetics	K (kinetics)	CFT (Clot formation time)	-
Time from 2 to 20 mm clot firmness	(1–4 min.)	-EXTEM CFT (46–148 sec)	
		-INTEM CFT (40–100 sec)	
Clot kinetics	Alpha angle	Alpha angle	-
Alpha angle	(53–67 deg)	-EXTEM angle (63–81 deg)	
		-INTEM angle (71–82 deg)	
Clot strength	MA (Maximum amplitude)	MCF (Maximum Clot Firmness)	-
		-EXTEM MCF ( 49–71 mm)	
	(55–73 mm)	-INTEM MCF (52–72 mm)	
		-FIBTEM MCF (9–25 mm).	
Clot elasticity	G (4.6-10.9 dynes/cm^2^)	-	-
Time until the beginning of fibrin formation	-	-	Son Act (85–145 sec)
Rate of fibrin formation	-	-	Clot Rate R1 (15–45 clot signal units/min)
Completion of fibrin formation	-	-	Peak (mm)
Index of fibrinogen conversion to fibrin	-	-	Time to Peak (540–600 sec)
Platelet-induced clot retraction	-	-	R3 (>2 mm/min)

*EXTEM reagent contains tissue factor, INTEM reagent contains ellagic acid, FIBTEM reagent contains platelet inhibitor.

Citrate-anticoagulated samples were analyzed consecutively for hemoglobin concentration, platelet counts, international normalized ratio (INR), activated thromboplastin time (aPTT), fibrinogen, d-dimer and antithrombin by standard methods at the Department of Medical Biochemistry at St. Olav University Hospital.

### Statistical analysis

Data are given as frequencies or means with standard deviation (SD). For the statistical analysis of the RoTEM variables, only the endpoint and not early values were used to ensure comparability because the TEG and Sonoclot instruments do not give early values. Repeated measures analysis of variance (ANOVA) was used for analysis of changes in continuous variables by time. If necessary to obtain a good model fit, logarithmic or rank transformation was performed. *p-*values < 0.05 were considered statistically significant in the ANOVA analysis. Linear correlations were evaluated by Pearson’s R. To correct for correlations calculated at multiple time points, correlations with *p*-values < 0.01 were considered significant. Statistical analysis was performed using the program SPSS for Windows, version 16.0 (SPSS Inc. Chicago, IL, USA).

### Protocol approval and patient consent

This was a prospective observational study, approved by the Regional Committee for Medical Research Ethics, Central Norway. All patients who were asked to participate in the study consented.

## Results and discussion

Patient demographics, medical data and data on surgical procedures are presented in Table 2.

**Table 2:** Patient demographics and data on surgical procedures (n = 35)

Age (years)	62.8 (10.3)*
Gender (male/female)	22/13
Weight (kg)	82.7 (13.4)*
Preoperative blood hemoglobin concentration (g/dL)	13.7 (1.4)*
Preoperative LMWH (yes/no)	7/28
Additive EuroSCORE	3.94 (2.70)*
Operation type	
CABG	23
Valve surgery	9
Combined CABG and valve surgery	3
Surgical time (min)	156 (46)*
Cardiopulmonary bypass time (min)	82 (36)*
Aortic cross-clamp time (min)	52 (29)*
Red cell transfusion (yes/no)	15/20
Fresh frozen plasma (yes/no)	8/27
Platelet transfusion (yes/no)	3/32
Desmopressin 30 μg/kg (yes/no)	6/29

*Values expressed as mean (SD).

Gender, preoperative LMWH, operation type, red cell transfusion, fresh frozen plasma transfusion, platelet transfusion and treatment with desmopressin are given as total number of patients in each category.

CABG = coronary artery bypass grafting, EuroSCORE = The European System for Cardiac Operative Risk Evaluation, LMWH = low molecular weight heparin.

All the analyzed conventional coagulation tests differed significantly with time (Table 3). There were significant changes with time for the TEG variables R (reaction time), K (clot kinetics), alpha (clot strengthening), MA (clot strength) and G (clot elasticity), mainly between the preoperative results and the results recorded 1 hour after surgery (Table 4). MA and G were also significantly different from baseline 24 hours postoperatively. The RoTEM variables INTEM CT (clotting time), INTEM CFT (clot kinetics) and FIBTEM MCF (clot strength) changed significantly with time. For the Sonoclot, only Son ACT values (time until fibrin formation) differed significantly with time.

**Table 3:** Conventional coagulation tests at the three time points (n = 35)

**Parameter**	**Baseline**	**1 h postoperatively**	**24 h postoperatively**
Hemoglobin (g/dL)	13.1 (1.5)	10.5 (1.4)**	10.2 (1.4)**
Platelet count^a^ (×10e9/L)	226 (49)	182 (58)**	165 (56)**
Fibrinogen (g/L)^b^	3.4 (0.8)	2.4 (0.5)**	3.3 (0.6)
d-dimer^b ^(mg/L)	0.6 (0.6)	0.8 (0.9)**	0.4 (0.4)**
Antithrombin (%)	92 (10)	68 (13)**	75 (15)**
INR^a^	1.1 (0.1)	1.2 (0.1)**	1.3 (0.1)**
aPTT ( seconds)	28 (2.2)	31 (11.5)*	27 (2.6)*

Values are expressed as mean (SD).

INR - International Normalized Ratio, aPTT - activated thromboplastin time.

^a^Logarithmic transformation.

^b^Rank transformation.

*p <0.05 compared to baseline.

**p < 0.001 compared to baseline.

**Table 4:** TEG, RoTEM and Sonoclot variables at the three time points (n = 35)

**POC-variables**	**Baseline**	**1 h postoperatively**	**24 h postoperatively**
**TEG**			
TEG R^a^ (min)	8 (2.1)	10 (2.6)*	8 (2.1)
TEG K^a^ (min)	2 (0.6)	3 (0.7)**	2 (0.5)
TEG alfa (deg)	61 (6.1)	56 (7.5)**	63 (5.6)
TEG MA (mm)	63 (4.3)	58 (4.9)**	61 (6.1)*
TEG G^a^ (dyn/sec)	9 (1.7)	7 (1.4)**	8 (2.1)*
**RoTEM**			
INTEM CT^a^ (sec)	165 (28.8)	179 (25.6)*	153 (22.5)
INTEM CFT (sec)	79 (32.4)	84 (23.0)	104 (36.2)**
FIBTEM MCF^a^ (mm)	15 (4.3)	12 (3.4)**	16 (9.4)
**Sonoclot**			
SonACT^a^ (sec)	167 (33)	221 (99)*	165 (23)

Values are expressed as mean (SD). For variable definitions, see Table 1.

^a^Logarithmic transformation.

*p <0.05 compared to baseline.

**p < 0.001 compared to baseline.

Correlations between the conventional coagulation tests and POC variables are given in Table 5. TEG MA (clot strength) and RoTEM MCF (clot strength) correlated with fibrinogen at baseline. Even though both TEG alpha (clot strengthening) and TEG G (clot elasticity) were correlated to fibrinogen 1 and 24 hrs postoperatively and TEG K (clot kinetics) was correlated to fibrinogen 24 hrs postoperatively, TEG MA (clot strength) was the only TEG variable that correlated with fibrinogen at all time-points. The RoTEM MCF (clot strength) variables and the Sonoclot Clot Rate (rate of fibrin formation) also correlated to fibrinogen at all time-points.

**Table 5:** Correlations between conventional coagulation tests and POC variables (n = 35)

**Coagulation test**		**1 h postoperatively**		**24 h postoperatively**	
	**POC variable**	***p-*** **value**	**R value**	***p*-value**	**R value**
Fibrinogen					
	**TEG**				
	TEG K	0.13	- 0.42	0.002	- 0.52
	TEG alpha	0.004	0.48**	0.001	0.53**
	TEG MA*	< 0.0005	0.76**	< 0.0005	0.63**
	TEG G	< 0.0005	0.78**	< 0.0005	0.67**
	**RoTEM**				
	EXTEM MCF*	< 0.0005	0.71**	0.001	0.58**
	INTEM MCF*	0.001	0.53**	< 0.0005	0.63**
	FIBTEM MCF*	< 0.0005	0.79**	0.003	0.50**
	**Sonoclot**				
	Clot Rate*	0.002	0.53**	0.008	0.47**
Platelet count					
	**TEG**				
	TEG MA	0.67	0.08	0.009	0.44**
	**RoTEM**				
	EXTEM MCF	< 0.001	0.53**	0.04	0.36
	INTEM MCF	0.04	0.36	0.002	0.52
aPTT					
	**Sonoclot**				
	Son ACT	<0.0005	0.86**	0.001	0.58**
	Clot Rate	<0.0005	**-** 0.67**	0.007	- 0.48**

For variable definitions, see Table 1.

*Statistically significant correlation preoperatively.

**P-values < 0.01 were considered statistically significant.

Platelet counts correlated with TEG MA (clot strength) 24 hrs postoperatively, and two different RoTEM MCF variables (clot strength) correlated with platelet counts either 1 hr or 24 hrs postoperatively. The only correlations with aPTT were with the Sonoclot variables Son ACT (time until fibrin formation) and Clot Rate (rate of fibrin formation) at 1 and 24 hrs postoperatively. None of the POC variables correlated with INR.

Four patients needed a surgical reexploration, due to persistent postoperative bleeding (total bleeding 800–2690 mL). These patients were transfused with red cell concentrates (6–10 units), platelet concentrates (0–3 units) and FFP (3–6 units). Two of these patients received an additional dose of tranexamic acid postoperatively. The decision of giving tranexamic acid postoperatively was empirical, as the results of the POC instruments were not available for the attending physician. None of the bleeding patients had abnormal baseline values for the TEG, RoTEM or for the coagulation tests. Preoperative Sonoclot Son Act (time until fibrin formation) results were abnormal in three of these patients. However, pathological Son Act results were found in the majority of the patients preoperatively. Only one patient showed pronouncedly reduced RoTEM FIBTEM MCF (maximal clot firmness), plasma fibrinogen and TEG alpha (clot strengthening) values in the early postoperative period.

As expected, all the conventional coagulation tests showed significant changes following cardiac surgery. This was mirrored in changes in all the TEG variables, some of the RoTEM variables, and only one of the Sonoclot variables. The changes pointed towards a more hypocoagulable state, consistent with dilutional coagulopathy and consumption of platelets and coagulation factors occurring during CPB [7,8]. In most patients, the changes reversed to baseline values within 24 hours after surgery. In contrast to other studies [9,12], we did not observe increased fibrinolysis, which may be explained by the routine use of a prophylactic antifibrinolytic agent.

Several previous studies [22,25-28] do not support the routine use of POC instruments prior to surgery to predict the need for transfusion, due to the high number of false positive and false negative results obtained. However, rapid assessment of the cause of bleeding if it ensues in the postoperative period is essential to provide adequate treatment to the patient. Alterations in coagulation usually occur during CPB, mainly due to hemodilution and platelet activation caused by the contact with plastic surfaces. For a POC instrument to be useful, it should be sensitive enough to indicate such alterations in hemostasis in a timely fashion, and the data from 1 hr postoperatively may be the most relevant when comparing the instruments. In that regard, a variant of the TEG analysis, Rapid TEG, where tissue factor is added to kaolin for activation of the extrinsic pathway has shown promising results. Several studies have demonstrated that Rapid TEG is useful as the results are available in about 20 minutes, correlate well with conventional coagulation tests, and may be used to predict transfusion of red cells, plasma and platelets [29,30].

From our study, TEG seemed to be the more sensitive of the POC instruments and the Sonoclot was the least sensitive. None of our patients had markedly abnormal hemostasis and we cannot exclude that the instruments would have given more comparable results in patients with severe hemostatic disturbances.

Previous comparative studies to assess the interchangeability between TEG and RoTEM have been reported [24,31]. Even if TEG and RoTEM are very similar instruments, there are some differences between them. The RoTEM instrument analyses each parameter in a separate channel while in the TEG, all the parameters are analyzed simultaneously. In the TEG, the cup oscillates around the metal pin, whereas the pin moves around the cup in the RoTEM, conferring more stability against vibrations. The TEG uses kaolin and the RoTEM uses tissue factor (EXTEM) or the contact activator ellagic acid (INTEM) as activator of the coagulation process. The different plastic composition of the reaction cups has been pointed out as a possible additional factor for the lack of interchangeability between the two instruments [17]. All these differences may explain the divergent results obtained from the two instruments in our study and reported by other authors.

From our study, it seems that all three POC instruments may be equally useful to quantify changes in fibrinogen. The correlations found between various POC variables and fibrinogen concentrations are consistent with previous studies [20,32-34]. The importance of fibrinogen in promoting sufficient coagulation and hemostasis and as a contributor to the clot strength has lately been underscored [17]. Reduced fibrinogen levels during CPB identify patients at risk for post-operative bleeding and high transfusion requirements [13].

No correlations were found between INR and the TEG variable R (reaction time), the RoTEM CT (clotting time) or the Sonoclot Son ACT (time until fibrin formation), even if these three parameters all measure the time to the first fibrin formation. The reason for the discrepancy for TEG R might be the different activators used; kaolin for TEG and tissue factor for INR. For the other fibrin time measures, the reasons for the discrepancies are unknown. We found that the Sonoclot variables Son ACT and Clot rate (rate of fibrin formation) correlated significantly with aPTT. This is as expected, as aPTT is usually prolonged in hypocoagulable states, where a low Clot Rate can be found.

Preoperative platelet counts were within normal ranges in all the patients. None of the POC tests showed correlations with platelet counts at both time points in the postoperative period. The explanation may be that the POC instruments rather assess platelet function than platelet counts even if they may also indicate when platelet counts are too low to provide adequate hemostasis. From this point of view, the POC instruments may be more clinically useful than standard platelet counts.

Even if the TEG seemed to be more sensitive to detect hemostatic changes than the two other POC instruments, its use still carries potential problems. The references for the manufacturer’s proposed normal values for the TEG values are very limited and not well validated [34,35]. The manufacturer’s technical manual does not contain information about factors that may influence the results, such as gender, age or underlying medical conditions. In one study in 118 healthy blood donors [35] the authors found that 18.6% of the donors had at least one abnormal TEG parameter according to the manufacturer’s reference values, with the greatest differences involving the R (reaction time), alpha (clot strengthening) and MA (clot strength) parameters, even if none of the volunteers had abnormal routine coagulation assays. The authors recommended that each institution should determine its own reference values before adopting TEG. Concerns regarding blood sample stability and storage conditions have also been raised [17].

Laboratories should establish standardized procedures to minimize variability. To solve the mentioned problems the International TEG-RoTEM Working Group has been created to try to standardize TEG and RoTEM and to establish protocols to ensure accurate results [36]. Until better standardization is possible and the knowledge about factors influencing the results is increased, the routine coagulation tests will have to remain the gold standard for assessment of many aspects of hemostasis.

The main limitation of this study is the small number of patients. In addition, there was some missing data for the RoTEM analysis, caused by short-time supplier problems (n = 1-4). However, our results are consistent with previous findings from studies with separate evaluation of the TEG, RoTEM and Sonoclot instruments in cardiac surgery. Our study was not designed to study clinical outcomes in relation to the POC tests, which would require a much larger patient population.

## Conclusions

In conclusion, TEG and RoTEM can be used to detect postoperative hemostatic changes following cardiac surgery, whereas the Sonoclot seems to be less suitable, at least in patients without grave hemostatic changes. Variables from TEG, RoTEM and Sonoclot may be useful to monitor fibrinogen levels. Further studies are necessary to establish adequate reference values for different patient groups and to standardize these assays to achieve reliable results.

## Competing interests

The authors declare that they have no competing interests.

## Authors’ contributions

AE: Literature search, study design, patient enrolment, data collection and TEG, RoTEM and Sonoclot analysis, drafting of manuscript. RS: Study design, critical revision of manuscript. VV: Statistical analysis, critical revision of manuscript. HP: Study design, Sonoclot signature readings, critical revision of manuscript. All the authors read and approved the manuscript for publication.

## Pre-publication history

The pre-publication history for this paper can be accessed here:

http://www.biomedcentral.com/1471-2253/14/80/prepub
